# Synthesis and performance evaluation of ZnO/CdS photoanodes with copper sulfide (Cu_2_S) and carbon counter electrodes

**DOI:** 10.1038/s41598-024-74687-9

**Published:** 2024-12-30

**Authors:** Pooja B. More, Chaitali V. Jagtap, Vishal S. Kadam, Mu. Naushad, Nithesh Naik, Pavan Hiremath, Habib M. Pathan

**Affiliations:** 1 Advanced Physics Laboratory, Department of Physics, Savitribai Phule Pune University, Pune, Maharashtra 411007 India; 2 Department of Chemistry, College of Science, King Saud University, P.O. Box 2455, Riyadh, 11451, Saudi Arabia; 3 Department of Mechanical and Industrial Engineering Manipal Institute of Technology, Manipal Academy of Higher Education, Manipal, Karnataka 576104 India

**Keywords:** Counter electrode, Compact layer, Electrolyte, ZnO films, Cu_2_S films, Energy science and technology, Materials science, Nanoscience and technology, Optics and photonics, Physics

## Abstract

The present study demonstrates the synthesis of compact ZnO layers using CdS sensitized on ZnO as a photoanode with copper sulfide (Cu_2_S) and carbon as a counter electrode (CE). In this study, a compact ZnO layer was fabricated using the simple and low-cost successive ionic layer adsorption and reaction (SILAR) method, and Cu_2_S CE films were synthesized using the chemical bath deposition method. Various characterizations, such as X-ray diffraction (XRD) and X-ray photoelectron spectroscopy (XPS), confirmed the formation of ZnO and CdS sensitizations on the ZnO . UV-visible spectroscopy revealed that the bandgaps of the ZnO and Cu_2_S films were 3.2 and 1.3 eV, respectively. Furthermore, the morphology of the ZnO films was optimized by varying the number of SILAR cycles. Scanning electron microscopy revealed the formation of a nanorod compact layer (CL) and the porous nature of the ZnO photoanode films. However, the porosity increased with the number of SILAR cycles. Various parameters, such as the current density, voltage, fill factor, and efficiency, were measured using the J-V characteristics. The highest 0.85% efficiency was achieved by using the ZnO compact film with 30 SILAR cycles for the Cu_2_S CE. Furthermore, the study revealed that the Cu_2_S counter electrode had a higher electrocatalytic response than the carbon CE.

## Introduction

The rise in population, industrialization, and civilization all contribute to an increase in energy demand. Currently, the world’s energy needs are met using conventional energy sources such as coal and fossil fuels. Nevertheless, the high consumption of these energy sources results in a large amount of toxic gases in the climate, causing a greenhouse effect. Consequently, there is an immediate need to develop renewable energy sources to control the energy crisis^1–3^. Among various renewable energy sources, solar energy is the most favorable owing to its environmentally friendly nature^4^. Solar cells are photovoltaic (PV) devices that directly convert solar energy into electricity. In the future, there is no doubt that the constant utilization of solar energy will be required to meet the rising energy demand^5^.

In the past few decades, quantum dot-sensitized solar cells (QDSSCs) have attracted attention as a promising alternative to dye-sensitized solar cells (DSSCs)^6^. However, the photoconversion efficiency of DSSC reported so far is 28.9%, which is higher than QDSSC^7^. However, DSSC are expensive because of the higher cost of the dye compared to QDSSC^8^. Another important parameter is the tuning of the bandgap during the sensitization of the QDs in a QDSSC. By altering the size of QDs, their absorption inside the quantum confinement regime can be adjusted^9^.

On the other hand, the theoretical studies have estimated that QDSSC have a substantially greater power conversion efficiency (44%)^10^. However, the experimentally reported photoconversion efficiency of QDSSCs is still lower than the achievable theoretical efficiency. Quantum dots (QDs) possess distinct properties, such as tunable bandgaps, high extinction coefficients, and rapid charge separation, making them suitable for solar applications^11^. Various QDs such as cadmium sulfide/selenide (CdS/Se)^12^, lead sulfide/selenide PbS/Se^13^, and zinc sulfide (ZnS)^14^ can be utilized as potential sensitizers for QDSSCs. In addition, QDs can be synthesized by various methods, such as successive ionic layer adsorption and reaction (SILAR)^15^ and chemical bath deposition (CBD)^16^.

For the fabrication of sensitized solar cell devices, ZnO is a suitable candidate as a photoanode with higher thermal stability, higher mobility for excited photoelectrons, and lower cost than TiO_2_. Several materials act as counter electrodes in QDSSCs, including platinum^12^, PbS^13^, Cu_2_S^17^, and carbon^6^. In addition, the Cu_2_S counter electrode (CE) has attracted considerable attention owing to its conductivity, excellent catalytic activity against the polysulfide redox couple, nontoxicity, low cost, and optimal bandgap^14^. Therefore, Cu_2_S is suitable for various applications such as solar devices, superconductors, ion batteries, and sensors^18,19^. Various deposition methods have been used to synthesize Cu_2_S thin films. Despite this, the CBD method has various benefits like low cost, low pressure, and temperature reaction, large areas deposition, etc., over other deposition techniques such as vacuum evaporation, sputtering, and spray pyrolysis^20–23^. Therefore, polysulfide, carbon, and Cu_2_S are suitable as electrolytes and CE, respectively, for the fabrication of CdS sensitized solar cells, and are utilized in the present study^10^. The FTO/ZnO interface plays a crucial role in the fabrication of superior quality solar-cell devices. Therefore, a compact layer of ZnO is beneficial as it decreases the electron transfer from FTO to the electrolyte and reduces the interfacial resistance, which leads to an improvement in the performance of QDSSC^24^. Moreover, the ZnO compact layer forms a potential barrier between the FTO and the porous ZnO layer, which leads to an improvement in the electron transport across the mesoporous layer by enhancing the connectivity among the ZnO particles. Previously many research groups have studied ZnO compact layer to study their physicochemical properties and different applications^25^. Nirmal et al. studied the effect of ZnO NR growth on the variation in the seed layer thickness deposited on PEN substrates, which affects the rod diameter, density, and alignment^26^. Wang et. deposited Cu_2_S on FTO using the electrodeposition method, which is a suitable alternative for Cu_2_S/brass and Pt–CEs^27^. Jeong et al. reported an improvement in a CdSe sensitized based Cu_2_S CE deposited on conducting FTO substrate using drop casting or spin coating gives 2.1% efficiency^28^. SILAR technique stands out for producing uniform, high-quality ZnO films at low temperatures, suitable for applications requiring precise control over film thickness and surface morphology. Compared to techniques like chemical vapor deposition or sol-gel processes, SILAR is more scalable and cost-effective, making it suitable for large-scale production^29^. The study further optimized the growth of ZnO compact films using the SILAR method from 10 to 50 cycles. A comparative study of the electrocatalytic characteristics of the Cu_2_S and Carbon CEs based on the influence of the compact layer and cell was performed. Cu_2_S exhibits a higher photovoltaic efficiency, ranging from 0.18 to 0.85%, when compared to carbon CE.

## Experimental

**Fig. 1:**
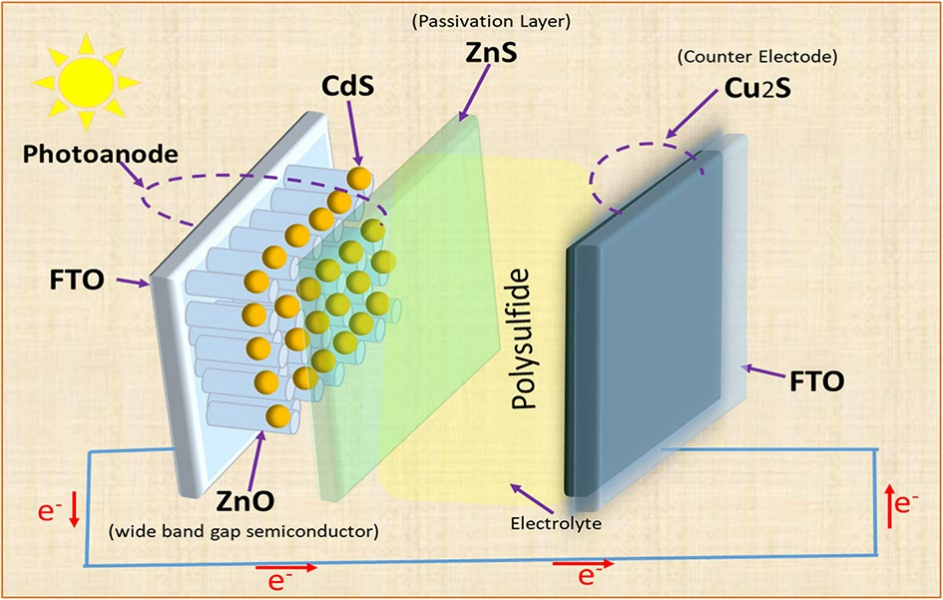
Working principle of QD-sensitized solar cell.

All reagents used in the experiments were of analytical grade and used without further purification. All were purchased including FTO glass (Sigma Aldrich), zinc sulfate (HPCL, India), Cetyl Trimethyl Ammonium Bromide (CTAB) (SRL), ammonia (HPLC, India), Cadmium Nitrate (SRL), ZnO powder (SRL), Ethyl Cellulose (SDFCL, India), terpineol (KPS Ltd., India), acetylacetone (HPCL, India), sodium sulfide hydrate (Sigma Aldrich), triethanolamine (HPLC, India), Copper Chloride (Merck), thiourea (Thomas Baker), and double-distilled water (DDW). Figure 1 shows a schematic representation of the working principle of the CdS-sensitized solar cell.

### Preparation of the zinc oxide compact layer

**Fig. 2:**
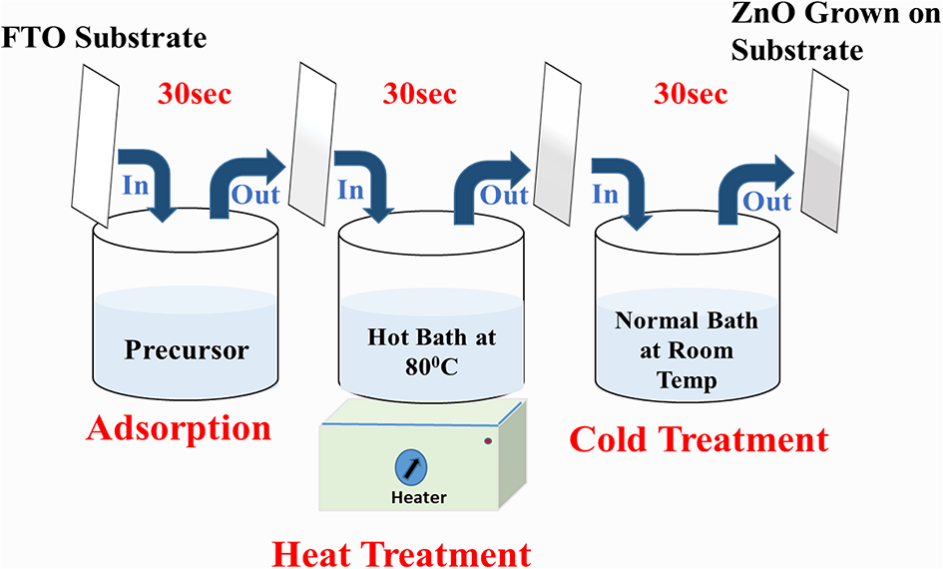
Synthesis of mesoporous ZnO compact film by using three beaker SILAR system.

The compact ZnO layer (CL) was fabricated using the CTAB-assisted SILAR method. In this synthesis process, 0.25 M CTAB and 0.1 M Zinc Sulphate [ZnSO_4_] were dissolved in DDW under stirring at room temperature for 15 min. Ammonia was added to the solution with continuous stirring until the precipitation was clear. A beaker system was used for SILAR deposition of the ZnO CL. In the First beaker, the prepared precursor solution was taken for a hot water solution maintained at 80 °C, and the third beaker was a normal bath of DDW at room temperature. A schematic representation of the ZnO compact film preparation using the SILAR method is shown in Fig. 2, where a dipping time of 30s was kept constant in each beaker for way varying cycles from 10, 20, 30, 40, and 50 cycles. Subsequently, the films were dried for 30 min in an incubator at a temperature of 60 °C and then annealed for 1 h at 450 °C in a muffle furnace.

### Fabrication of zinc oxide photoelectrode

ZnO films were fabricated on a pre-prepared ZnO CL using ZnO paste consisting of ZnO powder, ethyl cellulose, terpineol, and acetylacetone. The ZnO powder was ground with ethyl cellulose with the corresponding addition of Ethanol, Terpineol, and Acetyl Acetone to obtain a homogeneous slurry. This slurry was then used to fabricate photoelectrodes using the doctor-blade technique. The fabricated films were dried overnight at 60 °C in an incubator and annealed at 450 °C for 1 h. The as-annealed photoelectrode was used for CdS sensitization.

### Sensitization of CdS on ZnO film

For deposition of Cadmium Sulphide (CdS) sensitization, 0.05 M cadmium nitrate [Cd (NO_3_)_2_] and 0.05 M sodium sulfide [Na_2_S] the solution was dissolved separately in ethanol and methanol respectively. The ZnO photoanode was immersed in a cationic precursor solution of cadmium nitrate for 30 s, causing the cadmium ions to be absorbed on the surface of the substrate to form a CdS layer. Subsequently, the substrates were immersed in ethanol for 30 s. The substrates were then immersed in an anionic precursor solution of sodium sulfide for 30 s. Sulfide ions react with the cadmium ions adsorbed on the substrate. Subsequently, the substrates were immersed in methanol for 30 s. Thus, one SILAR cycle of the CdS film deposition was completed. A sensitized CdS sample was prepared by completing six SILAR cycles and subsequently dried naturally^7,30^.

### Surface passivation

Surface passivation is important to enhance the rate of electron transfer in the photoanode and decrease electron recombination from the photoanode with the redox electrolyte^31,32^. Three SILAR cycles of ZnS layer loading were made possible by the surface passivation of the porous ZnO film, which was sensitive to CdS. Using 0.01 M Zinc Acetate and 0.01 M Na_2_S in DDW, SILAR dipping was carried out to prepare the ZnS layer on the CdS-coated ZnO surface, SILAR dipping was performed.The dipping time in Each solution was dipped for 30s.

### Preparation of copper sulphide (Cu_2_S) film

The Cu_2_S CEs were deposited on FTO substrates via chemical bath deposition (CBD). Briefly, FTO substrates with a sheet resistance of 20.5 Ω cm^-2^ were washed with acetone, ethanol, and DDW for 20 min. Initially, the Copper chloride precursor was used as a source of Cu^+^ ions in 25 mL DDW. Under continuous stirring, 3–4 drops of TEA and 15% of ammonia solution which acts as the binding agent in the deposition to achieve the desired pH value. Then, 25 mL of thiourea solution was added to the solution as a source of S^-^ ions. After 40 min, the substrate was removed from the solution and a thin Cu_2_S layer was deposited on the FTO substrate. The precleaned FTO substrates were immersed vertically in the prepared solution. The colour of the solution changed from dark blue to dark brown during the deposition process, which shows the steps of the formation of Cu_2_S inside the bath solution.

### Fabrication of CdS-sensitized solar cell

ZnO/CdS working electrode (WE) with an active area of 0.35 cm^2^ was used with 45 μm spacers thickness at the edge. The Cu_2_S CEs were clamped together, facing the ZnO photoelectrode using binder clips on the opposite side. A drop of polysulfide electrolyte was added between them until no air bubbles were formed, and the CE and WE were clamped together. For comparison, Carbon CEs were also assembled into solar cells, and light of 1 sun intensity was induced such that light penetrated through the photoelectrode to the CdS adsorbed onto the ZnO layer.

### Characterization techniques

The XRD patterns of fabricated film onto FTO were determined using X-ray Diffraction (XRD) (Rigaku ‘‘D/B max-2400’’, Cu Ka = 0.154 nm). The morphologies of the films were determined by scanning electron microscopy (SEM, JEOL JSM 6360-A). A hydrophilic and hydrophobic analysis system (Ossila contact angle goniometer (L2004A1), Netherlands) with a high-resolution camera was used for the image capture. A UV-visible spectrophotometer (JASCO V-670) was used to determine the optical properties of the sensitized samples. The photovoltaic parameters of each cell were measured using a simulated solar stimulator (Enlitech Technology Co. Ltd. Taiwan). Electrochemical impedance spectroscopy (EIS) was performed using a potentiostat/galvanostatic instrument (IVIUM Vertex).

## Results and discussion

### Structural characterization

**Fig. 3:**
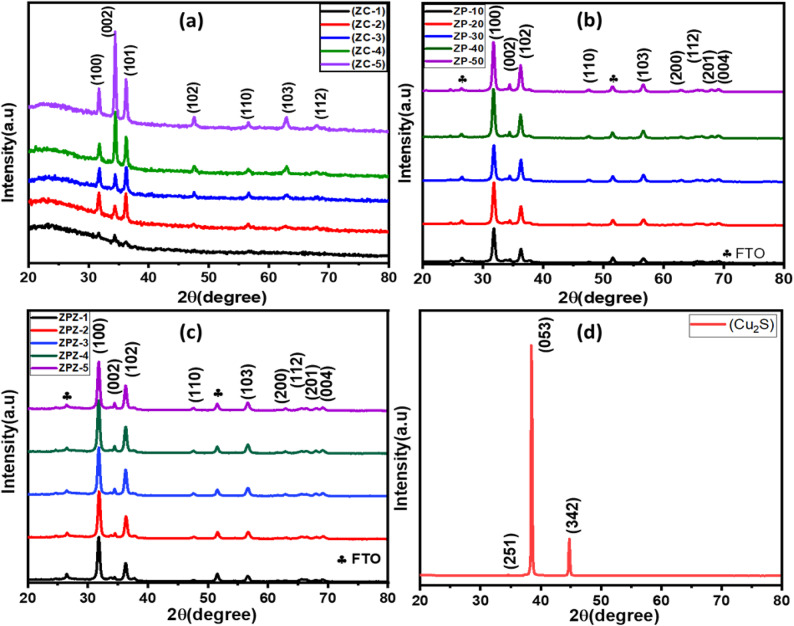
(**a**) X-ray diffraction pattern for ZnO compact film of 10-50cycles, (**b**) ZnO porous layer onto the compact layer, (**c**) ZnS passivated on CdS sensitized ZnO photoanode, (**d**) Cu_2_S CE film.

Figure 3a shows the XRD pattern of the compact ZnO layer from 10 to 50 cycles, designated ZC-1 to ZC-5. Figure 3b shows the XRD pattern of the ZnO paste deposited onto the compact for 10–50 cycles which is designated as ZP-1 to ZP-5. Furthermore, Fig. 3c shows the XRD pattern of ZnS passivation on CdS-sensitized ZnO photoanodes with 10–50 cycles, designated as ZPZ-1 to ZPZ-5. Figure-3d shows the XRD patterns of the Cu_2_S CE film. The obtained diffraction peaks in the XRD pattern Fig. 3a show that ZnO is crystalline and has a hexagonal wurtzite structure, matching the standard data according to JCPDS card No. 89–0511. The peaks at 2θ angle values 31.72^º^, 34.42^º^, 36.22^º^, 47.54^º^, 56.66^º^, 62.77^º^,67.98^º^ shows the plane orientations (100), (002), (101), (102), (110), (103), and (112) respectively. The major peak 2θ values of the ZnO film were 31.72^º^, 34.42^º^, and 36.22^º^ with plane orientations of (100), (002), and (101) planes, respectively. From Fig. 3a, it is evident that with an increase in the number of SILAR cycles, the orientation along the c-axis increases because of many aspects, such as the surface-energy viewpoint, the growth velocity along the c-axis direction will be the fastest as compared to any other growth facet^32^, nucleation sites on the ZnO layer increase, and the density and alignment level of the nanorods also increase^33^. Therefore, the growth of ZnO nanorods was highly aligned with the substrate. Finally, by increasing the reaction time and SILAR cycles, the intensity of the (002) plane also increased, which ensured that ZnO was grown preferentially along the c-axis^33,34^. Figure 3b, c show the XRD patterns of the ZnO porous layer using ZnO paste on the compact layer and ZnS/CdS/ZnO on the compact ZnO layer. From both XRD patterns, several peaks were observed at 2θ angles of 26.41º, 31.72º, 34.42º, 36.22º, 47.54º, 51.45º, 56.56º, 63.77º, 66.38º, 68.01º, and 69.12º, corresponding to the (100), (002), (101), (102), (110), (103), (112), (200), (201), and (004) plane orientations, respectively. The XRD pattern indicates the formation of a crystalline hexagonal structure. All peaks matched well with the standard data, according to JCPDS card No. 01-075-0576. In addition, the absence of impurity diffraction peaks indicated that the product was pure ZnO. Figure 3d XRD pattern for the Cu_2_S CEs film shows three peaks at 2θ values of 34.61°, 38.44°, and 44.74° to the corresponding plane orientations (251), (053), and (342), which matched well with JCPDS card No. 12–0227. Indicating the formation of the orthorhombic structure with chalcocite phase having cell parameters a = 11.88 Å, b = 27.32 Å, c = 13.49 Å, and α = β = γ = 90^º^.

### Optical studies: UV-visible spectroscopy

**Fig. 4:**
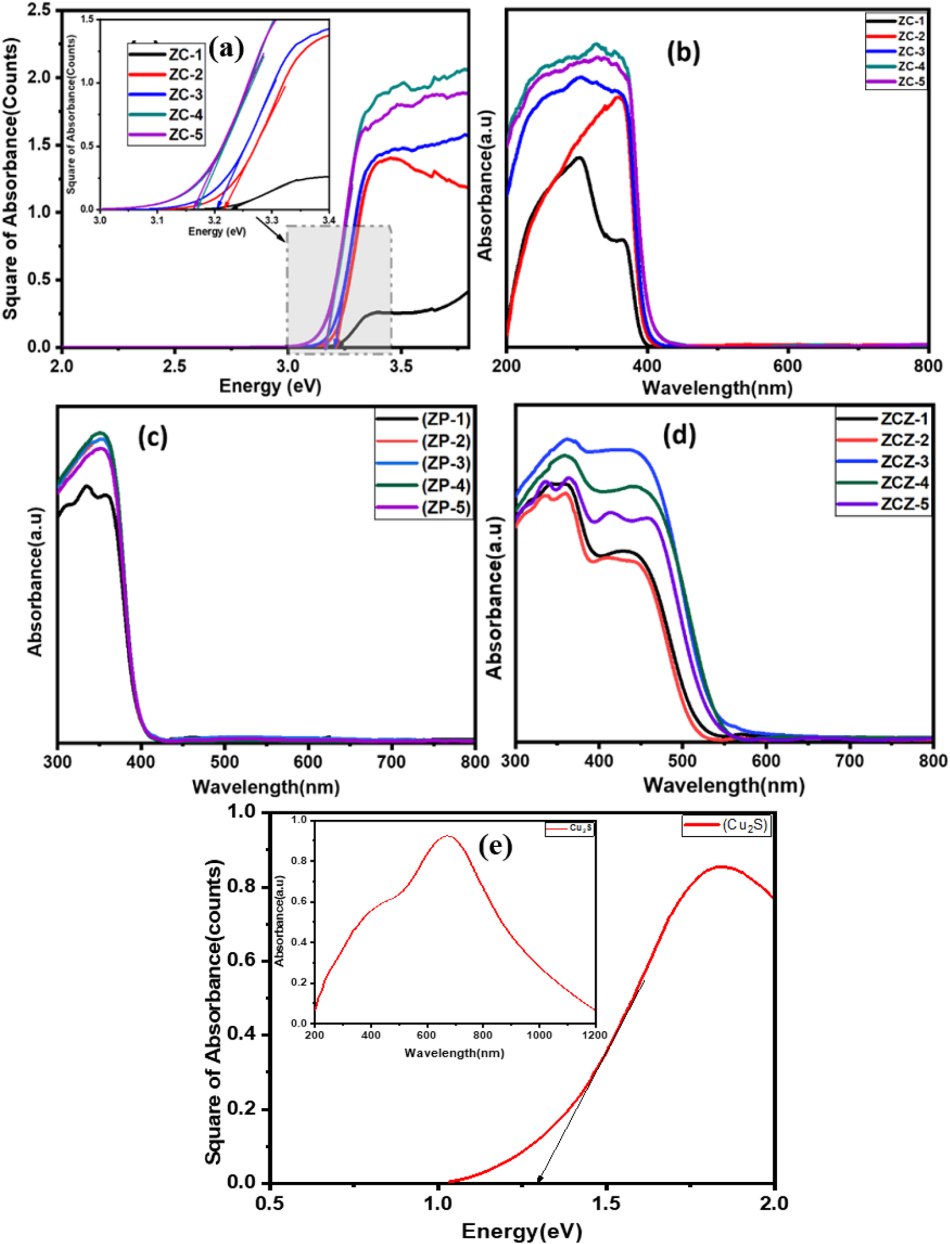
UV-visible spectra for (**a**) ZnO CL Tauc plot of 10–50 cycles (Inset zoom spectra), s (**b**) ZnO CL absorption spectra of 10–50 cycles, (**c**) ZnO porous layer onto CL, (**d**) ZnS passivation on CdS sensitized ZnO photoanode, (**e**) Cu_2_S CE film (Inset absorption spectra).

Figure 4 shows the (a) Tauc plot of ZnO compact from 10-50cycles (Inset zoom spectra), absorption spectra of (b) ZnO compact from 10-50cycles, (c) ZnO porous layer onto CL, (d) absorption spectra of ZnS passivation on CdS sensitized ZnO photoanodes, and (e) Tauc plot of Cu_2_S CE film (inset absorption spectra) obtained by UV–Vis spectroscopy. Figure 4a shows a Tauc plot of the ZnO CL from to 10–50 cycles, with the band gap of the films in the range of 3.23 to 3.16 eV. The variations in the bandgap is explained by the fact that during the growth of ZnO nanostructures, lattice deformation decreases, dislocation density changes, lattice strain changes, defects form in the ZnO, and electrons in the ZnO crystal lattice experience a periodic potential, leading to an change in the bandgap energy^35,36^. Ilican et al. reported that lattice strain affects the bandgap of ZnO by changing the interatomic spacing^37^. Ansari et al. studied ZnO grown in water to form more oxygen vacancies which lead to a decrease in its bandgap^38^. When the film thickness changed from 1.55 to 14.5 μm, a change in the bandgap energy of the ZnO thin film was observed. According to M. Ali et al., a decrease in bandgap occurs with an increase in film thickness^39^. Figure 4b The optical measurement result suggests that, when the film thickness increases and the absorption of films increases, more and more photons can be adsorbed on the surface of the material. Additionally, SILAR cycles can also affect the surface roughness, morphology, and defects, which influences the absorbance and bandgap energy^29,40^. Figure 4c shows the absorbance spectra of the ZnO paste deposited by the doctor blade method on the 10–50 cycles for the ZnO CL. Figure 4d shows the absorbance spectra of the CdS-sensitized ZnO photoelectrode, which serves as a sensitizer, enhancing the light absorption in the visible spectrum, which ZnO alone might not fully cover. The absorption region increases from the ultraviolet to the visible region of the spectrum owing to CdS sensitization^7^. Chou et al. reported the modification of ZnO nanowires with ZnO NPs; more deposition sites could be available for CdS QDs owing to the higher absorbance of ZnO NPs, because it has the highest surface^41^. The large increase in CdS sensitization in the 30 cycles of the ZnO CL is attributed to the higher surface area of the ZnO film available for adsorption. The surface area of ZnO decreases gradually due to the filling of CdS; therefore, a decrease in absorbance was observed in the later cycles (i.e. 40 and 50cycles)^42^. Figure 4e shows the absorption spectra measured in the wavelength range of (300–1100) nm for the Cu_2_S film. The deposited Cu_2_S thin film shows the optical band gap of 1.3 eV by using a Tauc plot. Therefore, the optical bandgap and XRD pattern indicate that the Cu-S system has a chalcocite structure^43^.

### Morphology and compositional study

**Fig. 5:**
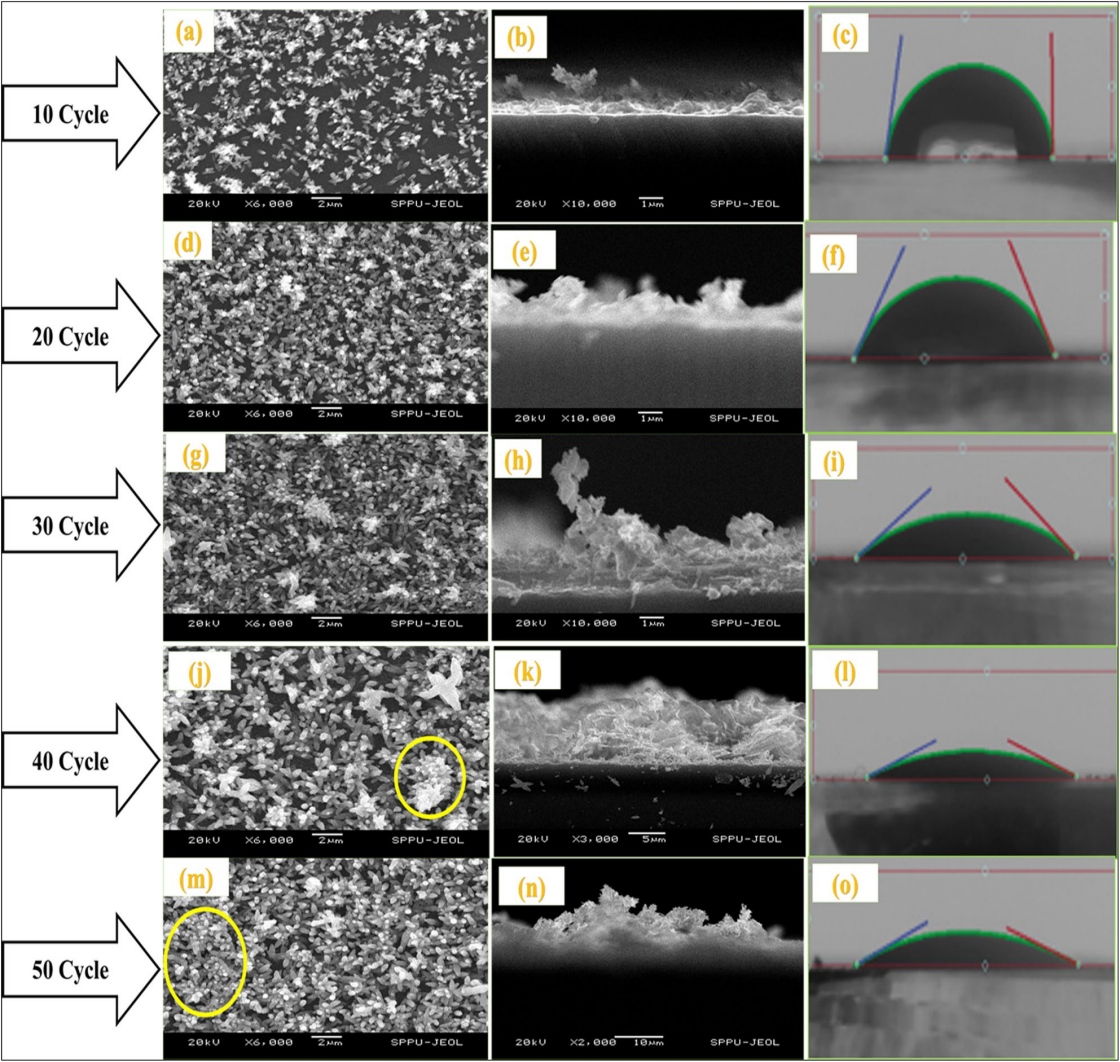
SEM images and cross-section images of ZnO compact films for various SILAR cycles with their Contact angles.

Figure 5 shows SEM and cross-sectional images of the ZnO compact films from 10 to 50 SILAR cycles. Furthermore, the contact angles were measured, as shown in Fig. 5. We observed the formation of nanorod structures that covered almost the entire substrate area. It is evident from the SEM images of the thin films that most of the nanorods were grown with some inclination, with a few exceptions of perpendicular growth on the substrate. With increasing number of SILAR cycles, the entire substrate was covered with nanorods, forming a dense network between them, as shown in Fig. 5. Incidentally, overgrowth was observed on top of the c-axis-oriented rods, which grew almost parallel to the substrate, as shown by the red circle in Fig. 5j, m. In short, as the number of SILAR cycles increased, the number of rods increased, and the inclination and film became more compact^44^.

**Fig. 6:**
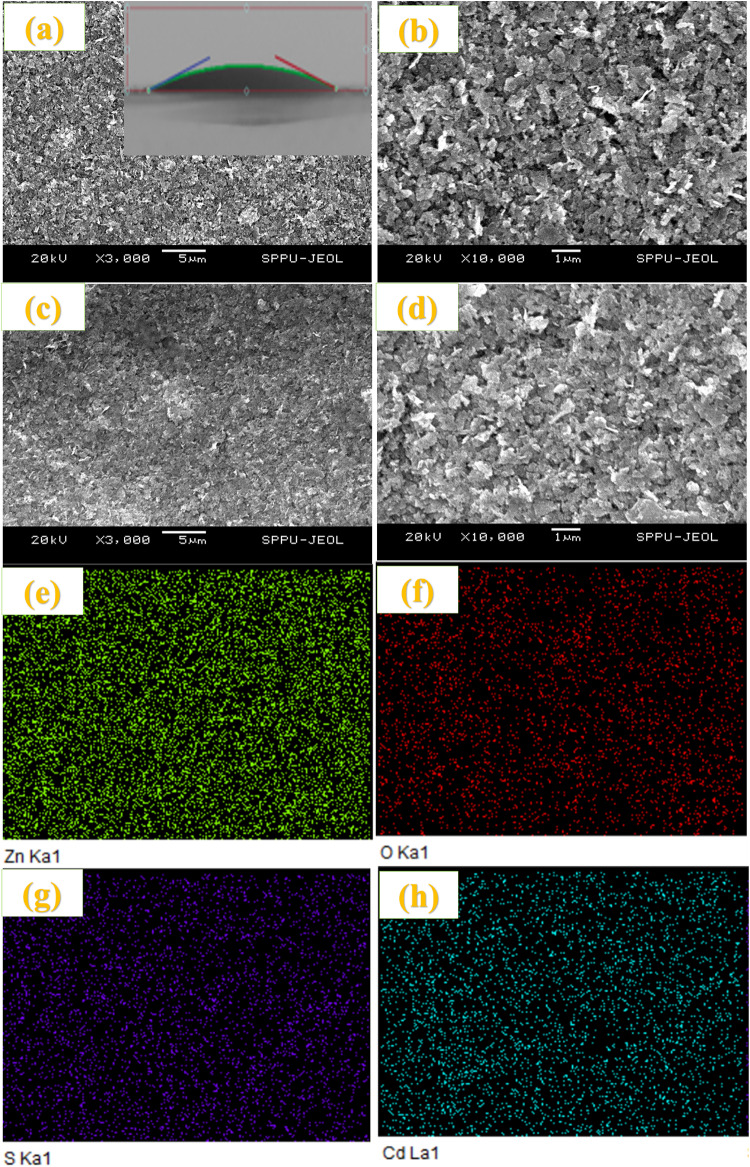
SEM images of (**a**,**b**) ZnO paste onto compact layer (Inset contact angle), (**c**–**h**) ZnS passivation on CdS sensitized ZnO photoanode and their mapping.

**Fig. 7:**
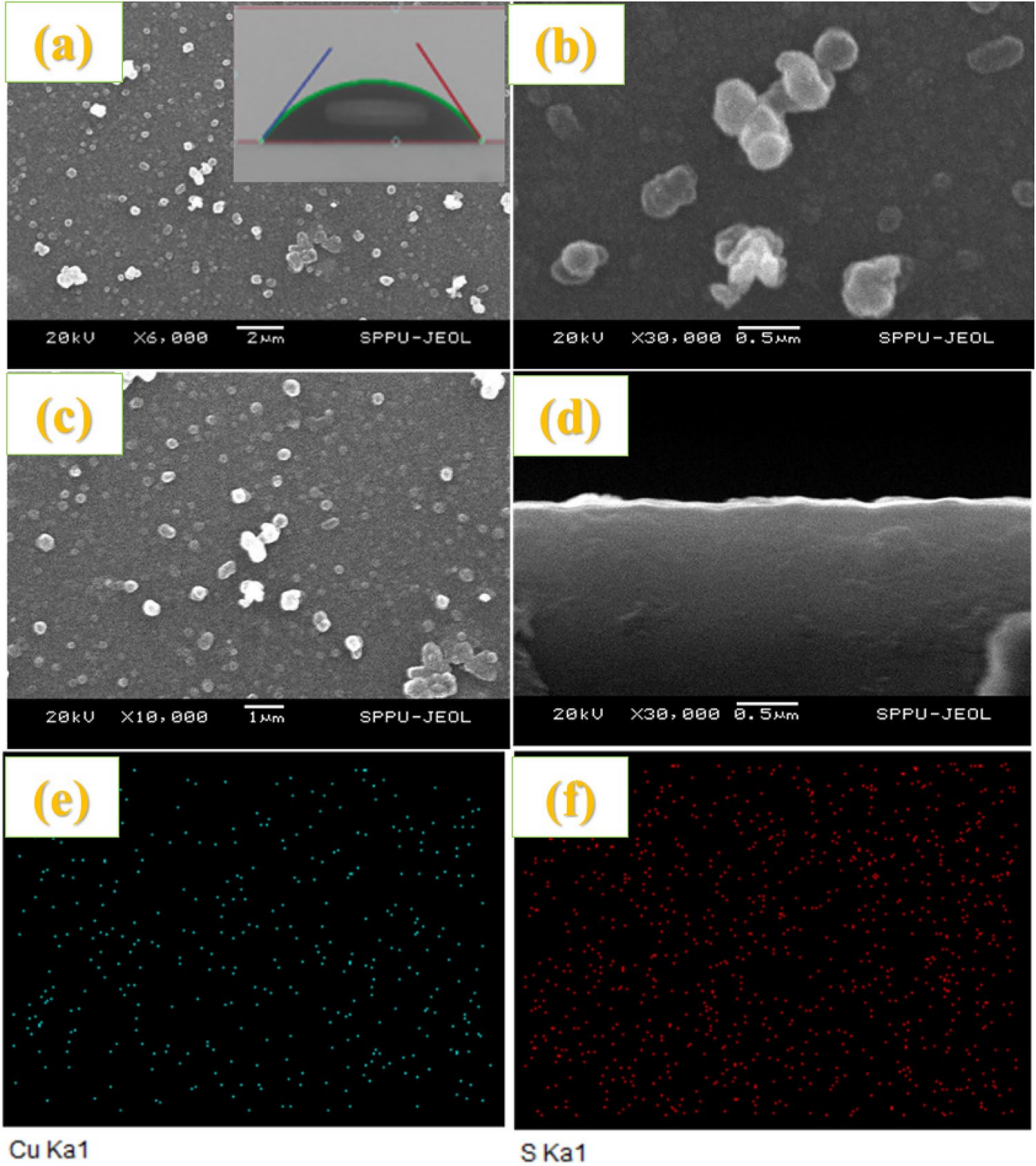
SEM images for (**a**–**c**) Cu_2_S CEs film with different magnifications (Inset Contact angle), (**d**) cross-section of Cu_2_S CEs, and (**e**,**f**) mapping of Cu_2_S film.

The approximate thickness of ZnO compact film varies for 10 to 50 SILAR cycles observed from ~ 1.55 to 14.5 μm. Figure 6a, b show the SEM images of the porous ZnO layer using the paste onto the CL. SEM images of (a, b) ZnO paste onto compact layer (Inset contact angle), (c-h) ZnS passivation on CdS sensitized ZnO photoanode and their mapping. This demonstrates the porous nature of the photoanodes. Figure 6c-h show the SEM and elemental mapping of ZnS passivation on the CdS-sensitized ZnO photoanode. Figure 7 shows the cross-sectional and morphological images along with the elemental mapping of Cu_2_S obtained using SEM measurements. Some clusters and irregular large particles were observed on the surface of the film, confirming the agglomeration of nanosized Cu_2_S crystallites into clusters^18^. The films were uniform and covered the substrate well.

### Contact angle analysis

**Fig. 8:**
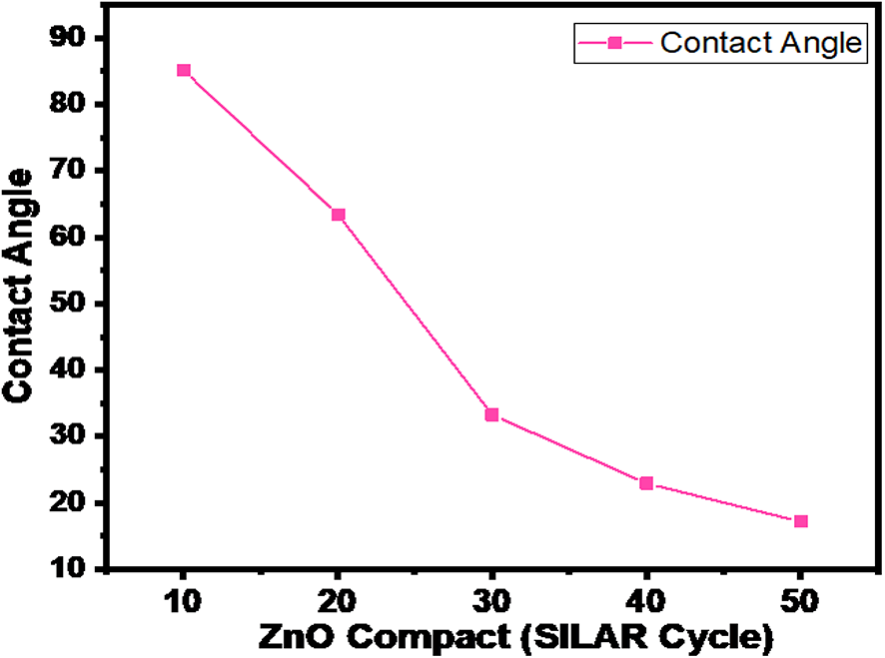
Contact angle concerning the SILAR cycles of ZnO film variations.

The hydrophobic and hydrophilic natures of the film were evaluated by contact angle measurements using a Drop Shape Analysis System (Ossila contact angle goniometer (L2004A1), Netherlands) with a high-resolution camera for image capture. Distilled water was used to evaluate film hydrophobicity. The average contact angle was calculated using the left and right contact angles, and the contact angle measurements of the ZnO compact films for 10–50 cycle variations are shown in Fig. 8. As the number of cycles increased, the contact angle decreased from 85.13° to 17.2°, indicating the hydrophilic nature of the compact ZnO films. The SILAR cycles of ZnO increased with a decrease in the contact angle, implying that the electrolyte could wet the surface very well, resulting in a greater contact area than that for the CL. This confirms that the porous nature of the material increases its hydrophilic behavior with varying SILAR cycles of ZnO to obtain an electrolyte and a more sensitive wet surface with greater contact^45^.

### X-ray photoelectron spectroscopy (XPS)

**Fig. 9:**
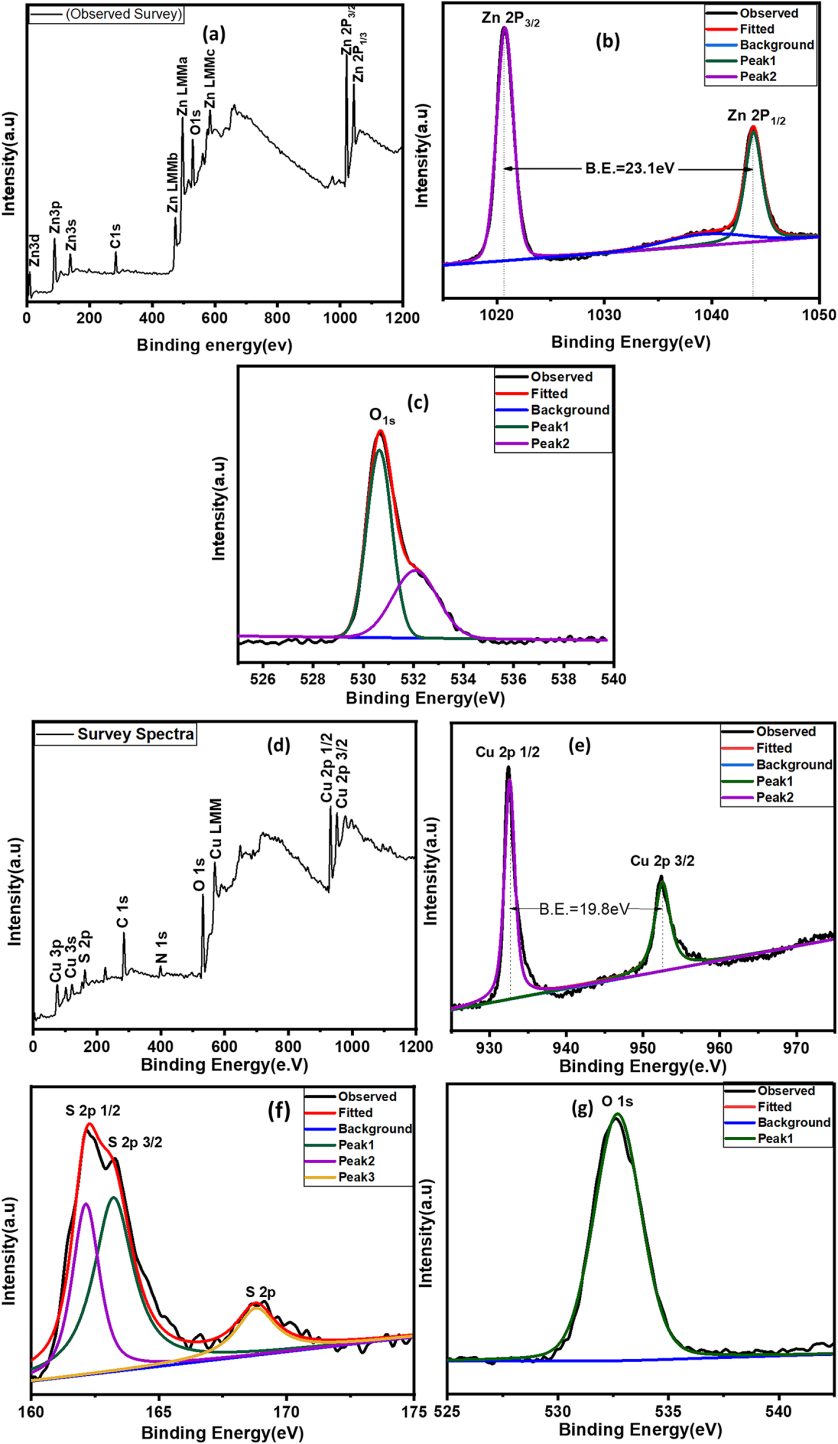

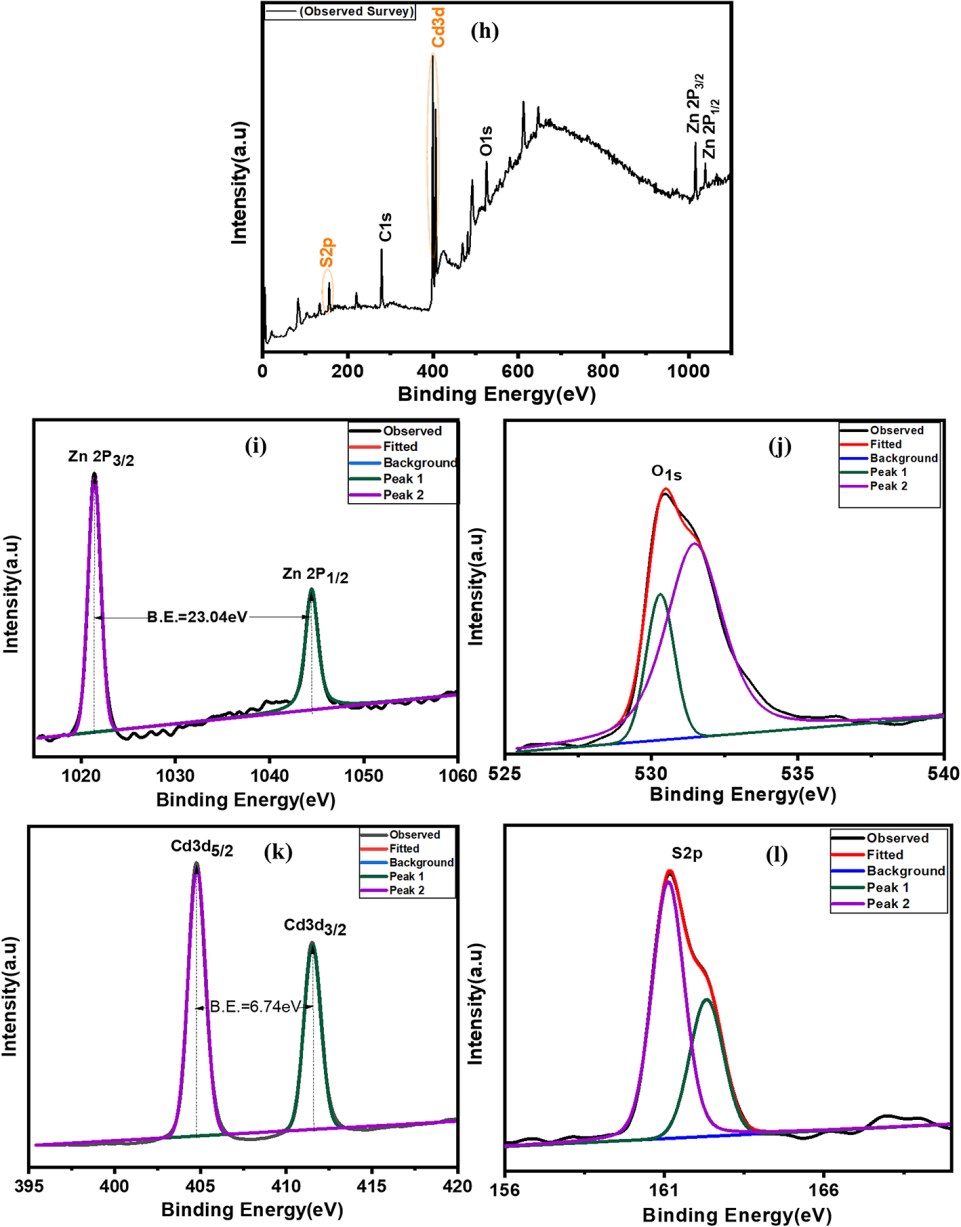
XPS spectra of (**a**–**c**) ZnO photoanode of 30 SILAR Cycle wide survey scan, Zn 2p Spectra, O 1s spectra, (**d**–**g**) Cu_2_S CE wide survey scan, Cu 2p spectra, S 2p spectra, O 1s spectra, (**h**–**l**) CdS sensitized on ZnO photoanode survey scan, Zn 2p Spectra, O 1s spectra, Cd 3d spectra, S 2p spectra.

X-ray photoelectron spectroscopy (XPS) of the ZnO photoanode is shown in Fig. 9a-c. XPS survey spectra indicated the presence of Zn, Oxygen, and Carbon were found on the surface of the ZnO film, and the binding energies were corrected by charge-shifting the C1s peak of graphitic carbon. Figure 9b shows the spectra for Zn which exhibits two fitting peaks for the Zn2p core level, situated at 1043.7 and 1020.2 eV, giving Zn2p_1/2_ and Zn2p_3/2_, respectively. The observed spin-orbit splitting, that is, the binding energy difference between Zn 2p_1/2_ and Zn 2p_3/2_ is approximately 23.1 eV for the ZnO photoanode^22^. Figure 9c shows that the O1s at 530.7 eV can be attributed to oxygen O^2−^ in the ZnO lattice. This indicates that no impurities were observed.

Figure 9d-g show the XPS spectra of the Cu_2_S CE film. A complete survey indicated the presence of Cu and S, and no other impurities were found on the surface of the Cu_2_S film. Figure 9e shows the XPS spectrum of the Cu2p core level. The peaks at a binding energy (B.E) of 932.3 and 952.1 eV were assigned to Cu 2p_1/2_ and Cu 2p_3/2_, indicating the presence of Cu^2+^ on the surface of the film. Spin-orbit splitting is the difference between the binding energies of the Cu 2p_3/2_ and Cu 2p_1/2_ levels at 19.8 eV^21^. The S 2p spectrum was fitted to the spin-orbit doublet assigned to binding energies of 162.0 and 163.2 eV for the S 2p_1/2_ and S 2p_3/2_ peaks, respectively. The characteristic S2p spectrum, with a binding energy of 168 eV, corresponds to SO_4_^2−^ anions^20^. The XPS spectra were corrected using the O 1s baseline, which was assigned a binding energy of 532.4 eV. These results confirm the formation of Cu_2_S, which is consistent with the XRD results.

Figure 9h-l shows the XPS spectra of the CdS-coated ZnO sample.The XPS survey which indicates the presence of cadmium (Cd), sulfur (S), and zinc (Zn) with carbon (C) and oxygen (O). The Zn2p_1/2_ and Zn2p_3/2_ peaks at about 1021.42 eV and 1044.46 eV confirm that the Zn exists mainly in the form of the Zn2 + chemical state on the sample surface. The O 1s main peak at 530.4 eV was assigned to metallic oxides. Figure 9k and l shows the two peak structure in the Cd 3d core level arises from a spin–orbit interaction with the Cd3d_5/2_ peak position at 404.52 eV and the Cd3d_3/2_ at 411.36 eV. The XPS binding energy of S 2p is 161.12 eV^46^.

### Transmission electron microscopy (TEM)

**Fig. 10:**
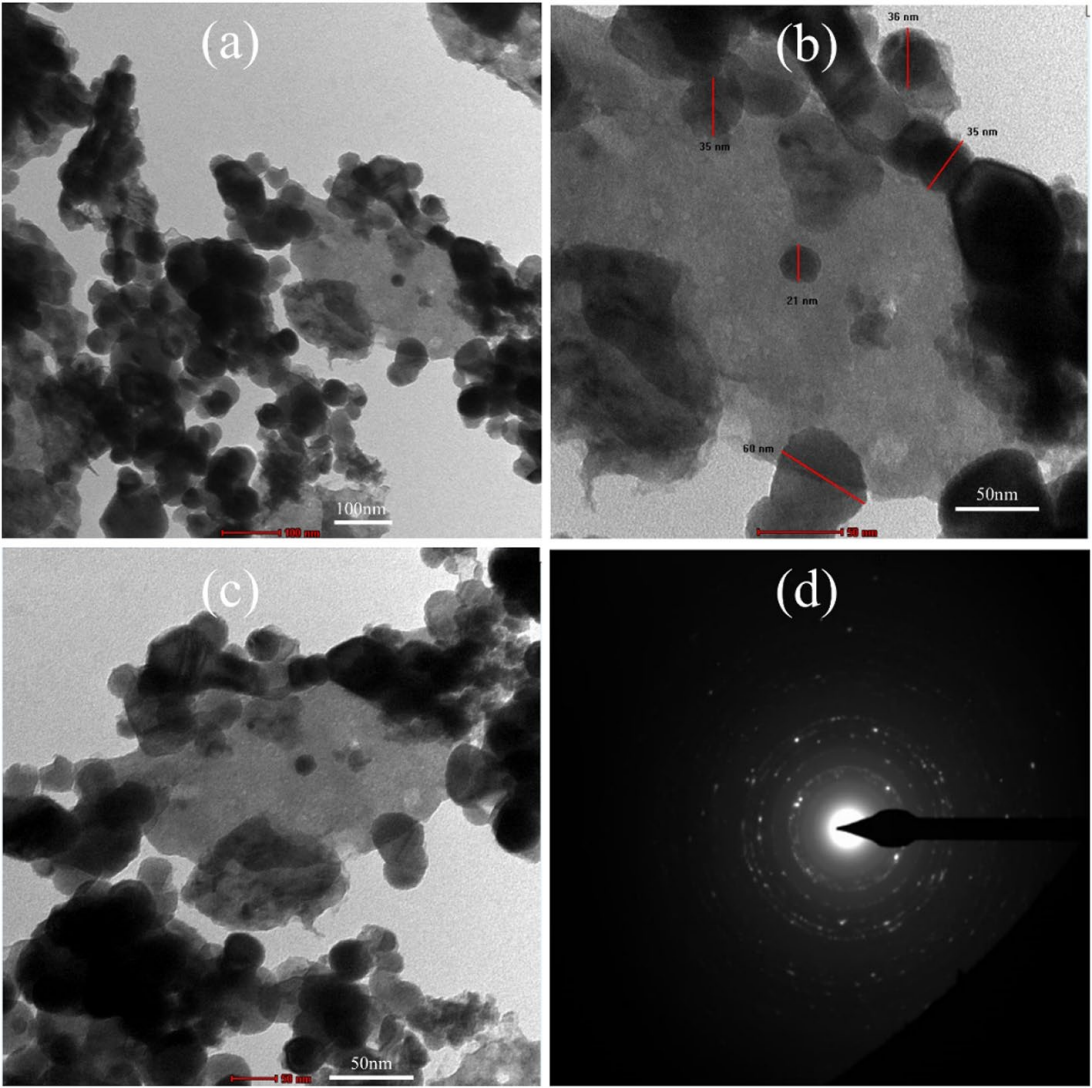
TEM images of (**a**–**c**) CdS sensitized ZnO photoanode, (**d**) SAED pattern of CdS sensitized ZnO photoanode.

The sensitization of CdS over the ZnO photoelectrode was further confirmed by transmission electron microscopy (TEM), as shown in Fig. 10a-c. A ZnO film was coated with CdS using the SILAR method. For the TEM images, the nanostructure architecture was scratched without disturbing the FTO substrate and was placed on a copper-coated carbon grid. The typical CdS grain size is between 20 and 35 nm which is greater than its radius; therefore, we can say it is nanoparticles (Fig. 10b). This micrograph of CdS nanoparticles is not distinctly observed because of the aggregation of CdS nanocrystals on the photoanode surface^47^. In addition, selected-area electron diffraction (SAED) was performed to investigate the crystalline characteristics of the ZnO/CdS thin film (Fig. 10d). The SAED pattern of the ZnO/CdS thin film revealed its polycrystalline nature. The bright diffraction spots with circular symmetry observed in the pattern were attributed to the ZnO crystal. Interestingly, the diffraction spots faded away in the images of the sensitized electrodes^48^.

### J-V characteristics

The performance parameters, such as short-circuit current density (J_sc_), open-circuit voltage (V_oc_), fill factor (FF), and power conversion efficiency (η), are listed in Table 1. The fabricated devices were tested under AM 1G simulated solar illumination with an intensity of 100 mW cm^-2^. The cell was tested at an active area of 0.35 cm^2^ kept constant for all the cells under measurement. For the Cu_2_S CEs, better results for the 30 SILAR cycles of ZnO resulted in V_OC_, J_SC_, FF, and E_ff_ were observed to be 0.6 V, 4.4 mA/cm^2^, 32%, and 0.85%, respectively, with a power of 152.6 µW. The performance parameters J_sc_, V_oc_.

**Table 1:** Photovoltaic performances of ZnO photoanode quantum dots-sensitized solar cell using Cu_2_S and Carbon films as counter electrode.

No SILAR cycles			Cathode	Current density Jsc (mA/cm^2^)	Short circuit current, I_SC_ or J_sc_ (mA)	Open circuit voltage, Voc (V)	Fill factor (FF)	Conversion efficiency (%)
ZnO compact layer	CdS	ZnS passivation Layer						
10	6	3	Cu_2_S-1	2.22	0.77	0.29	0.32	0.21
20	6	3	Cu_2_S-2	1.98	0.69	0.31	0.30	0.18
30	6	3	Cu_2_S-3	4.40	1.18	0.59	0.32	0.85
40	6	3	Cu_2_S-4	3.32	1.16	0.48	0.31	0.50
50	6	3	Cu_2_S-5	2.49	0.87	0.41	0.29	0.30
10	6	3	C1	0.31	0.11	0.19	0.18	0.01
20	6	3	C2	0.35	0.12	0.23	0.18	0.02
30	6	3	C3	0.30	0.105	0.25	0.15	0.01
40	6	3	C4	0.30	0.10	0.26	0.15	0.01
50	6	3	C5	0.47	0.16	0.27	0.19	0.02

**Fig. 11:**
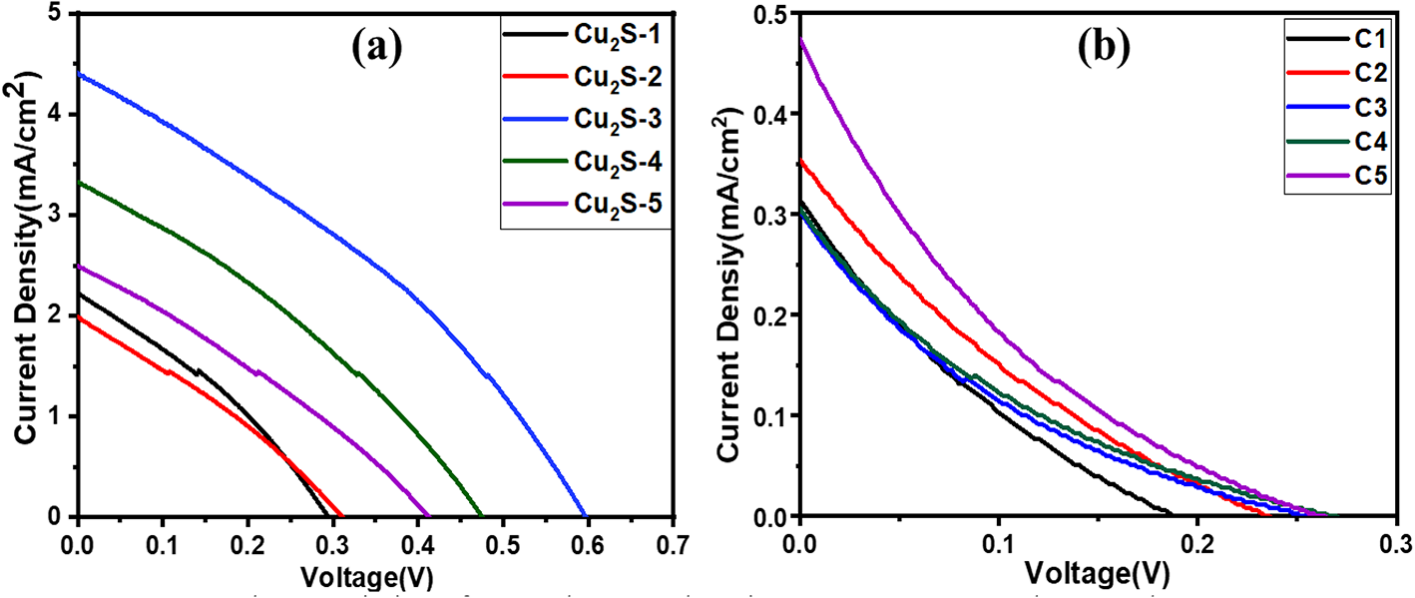
JV characteristics of ZnO photoanode using (**a**) Cu_2_S CEs and (**b**) Carbon CEs.

The FF and η values of the compact film after 30 SILAR cycles were better than those of the other SILAR cycles. As shown in Table 1, the Voc of Carbon CEs was lower than that of the Cu_2_S CEs. With the Cu_2_S CE, the maximum Voc is 0.59 V which is larger than that of the Carbon CEs. Figure 11 shows the photocurrent density-voltage curves of the device with two different CEs: (a) Cu_2_S CE and (b) Carbon CE. This was mainly due to the higher conductivity of the CE. These features led to the high catalytic activity of the Cu_2_S CE. This implies that Cu_2_S is superior to carbon as a counter electrode when a polysulfide electrolyte is used. Additionally, it evaluates every parameter and efficiency, with a summary from the recent literature included in Table 2. It is evident that the combination of a ZnO photoanode with a Cu_2_S CE has received less research attention to date.

**Table 2:** Studies from literature comparing the performance efficiency of QDSSC solar cells.

Sr. no.	Photoanode	QDs	Counter electrode	Jsc (mA/cm^2^)	Voc (V)	FF (%)	ɳ (%)	Refs.
1	TiO_2_	CdS	Cu_2_S	0.82	0.40	36	0.8	^23^
2	TiO_2_	CdS	Cu_2_S	3.70	0.280	28	0.29	^49^
3	TiO_2_	CdS	CuS	1.01	0.46	34	1.05	^50^
4	TiO_2_	CdS	CuS	0.71	0.58	48	1.38	^51^
6	TiO_2_	CdS	CuS	0.70	0.36	41	0.70	^52^
7	ZnO	CdS	Pt	0.52	3.16	-	0.68	^53^
8	ZnO	CdS	Carbon	0.36	0.14	21	0.06	^54^
9	ZnO	CdS	Carbon	1.51	0.48	23	0.25	^55^
10	ZnO	CdS	Cu_2_S	4.40	0.59	0.32	0.85	Present work

### Electrochemical impedance spectroscopy

**Fig. 12:**
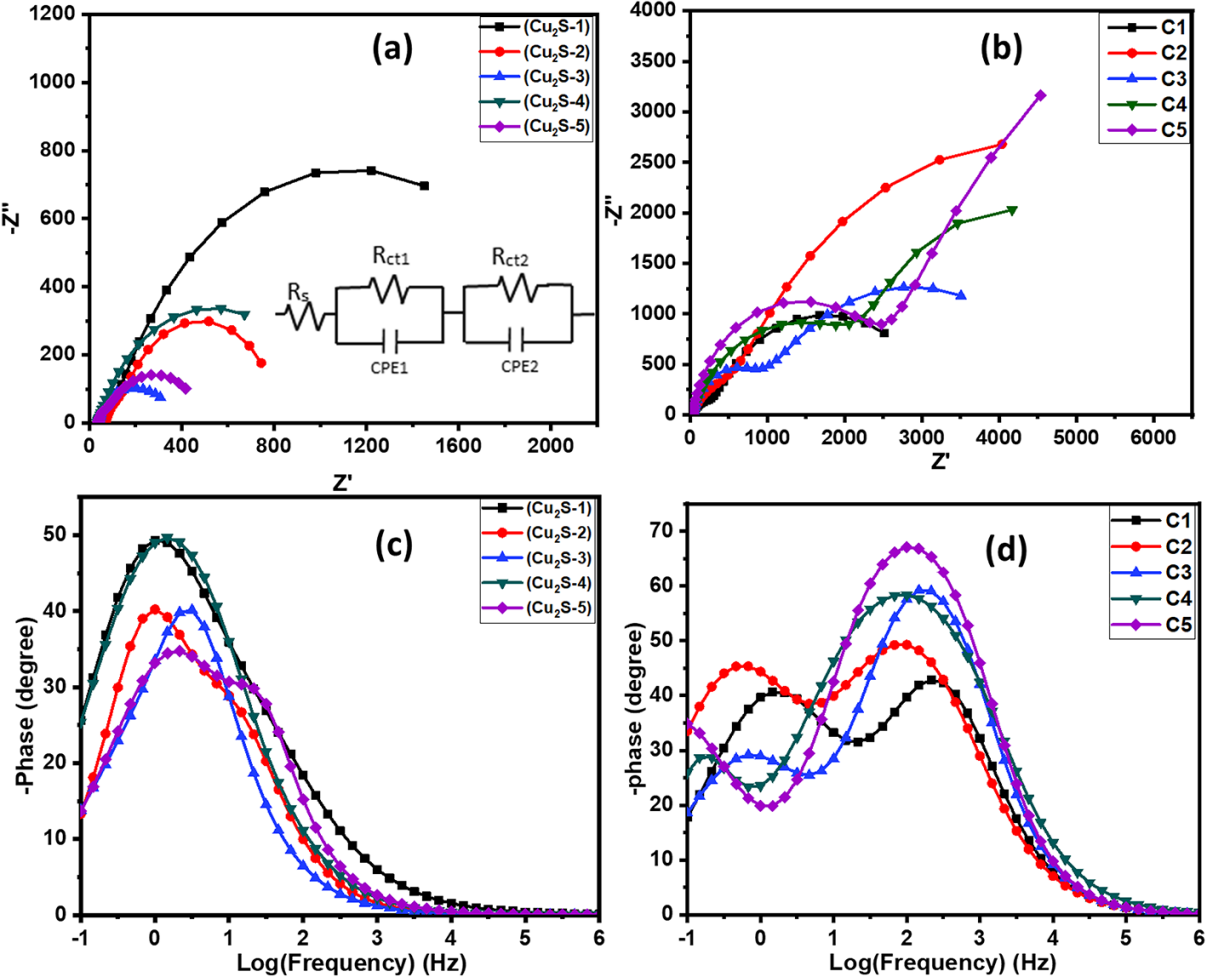
Nyquist plots of (**a**) Cu_2_S CEs, and (**b**) Carbon CEs. Bode plots of phase angles vs. log f impedance spectra of (**c**) Cu_2_S CEs, and (**d**) Carbon CEs.

Electrochemical impedance spectroscopy (EIS) was performed to study the electron recombination, transport mechanisms, and regeneration processes that occur in solar cell devices. EIS was carried out in a frequency range of 1 MHz to 0.1 Hz frequency range with a constant applied voltage. Figure 12 shows Nyquist and Bode plots obtained for the Cu_2_S and carbon CEs. Figure 12a, b show that the starting point corresponds to the sheet resistance (Rs) and that the two semicircles correspond to different charge-transfer mechanisms. The first arc in the high-frequency region (Rct1) is related to the electrolyte/counter interface, and the other arc in the low-frequency region (Rct2) is related to charge transport at the electrolyte/photoanode interface. In Fig. 12a it is observed that the 10 cycles of the ZnO CL with the Cu_2_S-1 CE show a semicircle of greater radius, indicating high charge carrier resistance with low charge carrier separation efficiency. However, 30 cycles of the ZnO CL with the Cu_2_S-3 CE showed the smallest semicircle radius, indicating the lowest charge transfer resistance and high charge carrier separation efficiency. The data obtained from EIS were fitted by the equivalent electric circuit shown in the inset of Fig. 12a, where CPE1 and CPE2 are constant-phase elements. The fitted parameters are listed in Table 3.

**Table 3:** Fitting parameters of EIS analysis for the fabricated devices obtained from EIS analysis (Nyquist and Bode plot).

Cathode	Rs (Ω)	Rct1 (Ω)	Rct2 (Ω)	Frequency, f_MAX_ (Hz)	Relaxation time (ms)
Cu_2_S-1	54.52	81.65	2006	1.46	0.109
Cu_2_S-2	71.17	91.8	655.5	1	0.15
Cu_2_S-3	38.93	66.2	274.2	3.16	0.05
Cu_2_S-4	35.78	1.781	989.5	1.46	0.109
Cu_2_S-5	38.17	23.36	442.4	2.15	0.07
C1	35.41	3007	230.6	215.4	0.73
C2	33.33	525.8	7745	100	1.59
C3	35.42	834.6	4054	146.7	1.08
C4	24.6	2421	3786	100	1.59
C5	30.84	2443	13,608	100	1.59

Figure 12c and d show the Bode plots of the phase angles vs. log frequency of the impedance spectra of the Cu_2_S and Carbon CEs. The mean carrier lifetime was calculated using the Bode plot. Equation (1) expresses the relaxation time constant as

1 $$\:{\tau\:}_{e}=\frac{1}{2\pi\:{f}_{max}},\:$$

where fmax is the peak frequency at the maximum phase, and $\:{\tau\:}_{e}$ is the relaxation time.

## Conclusion

In the present study, a ZnO nanorod layer was synthesized using the SILAR method and employed as a ZnO photoanode for a CdS-sensitized solar cell. Photoanodes sensitized by CdS nanoparticles were investigated and their photovoltaic characteristics were compared with those of carbon and Cu_2_S CE. XRD confirmed that ZnO was crystalline and had a hexagonal wurtzite structure, while Cu_2_S had an orthorhombic structure with a chalcocite phase. From the UV-visible study, the bandgaps of Cu_2_S and ZnO films are 1.3 and 3.1 eV, respectively. The absorption band increased from the ultraviolet to the visible region of the spectrum owing to ZnS passivation on the CdS sensitization. SEM revealed the nanorod morphology of the compact film and porous nature of the ZnO photoanodes. The TEM analysis indicated that the deposited CdS layer consisted of nanoparticles. A passivated layer of ZnS on the CdS-sensitized ZnO photoanode showed a noticeable improvement in the power conversion efficiency when a Cu_2_S CE was used instead of a carbon CE. The increase in η = 0.85% for ZnO/CdS/ZnS electrode employing a Cu_2_S CE.

## Data Availability

The datasets generated or analyzed during the current study are available from the corresponding author upon reasonable request.
